# Headache prevalence and impact among school-aged children in a Japanese town: the AMI-GRAINES study

**DOI:** 10.1038/s41598-025-30859-9

**Published:** 2025-12-03

**Authors:** Sumire Ishiyama, Akira Matsumura, Mikito Hayakawa, Yutaka Kohno, Eiichi Ishikawa

**Affiliations:** 1 Center for Medical Sciences, Ibaraki Prefectural University of Health Sciences, Ibaraki, Japan; 2 Department of Neurosurgery, Tsukuba Headache Center, Ichihara Hospital, Tsukuba, Japan; 3 Department of Stroke and Cerebrovascular Diseases, University of Tsukuba Hospital, Tsukuba, Japan; 4 Department of Neurology, Institute of Medicine, University of Tsukuba, Tsukuba, Japan; 5 Department of Neurosurgery, Institute of Medicine, University of Tsukuba, Tsukuba, Japan

**Keywords:** Children, Migraine, Pediatric headache, School absence, Disability, Health care, Neurology

## Abstract

Headaches are common in youth and can impact school and daily life. Data on headaches and care-seeking in Japanese schoolchildren are scarce. We investigated prevalence, impact, and consultation patterns. A school-based online survey was conducted between February and March 2025 among 3,766 students in seven elementary and three junior high schools in Ami-town, Ibaraki-prefecture, Japan. Two questionnaire versions were distributed: one for lower-grade students (grades 1–3) and one for upper-grade students (grades 4–9). The survey included items on headache frequency, triggers, associated symptoms, medication use, and consultation experiences. The prevalence of headaches was 29.1% in lower-grade and 37.6% in upper-grade students. The most common age of onset was 10 years. Students with Headaches that meet most of the diagnostic criteria for episodic migraine experienced more headache days and more school absences compared to students with other headache characteristics. Although nearly half of the students had taken medication, fewer than one-third had consulted a physician. Despite experiencing headaches, only a small number of children sought medical care, which may raise concerns about the potential overuse of over-the-counter medications. Therefore, collaboration between educational and medical institutions is essential to improve early recognition and intervention.

**Supplementary Information:**

The online version contains supplementary material available at 10.1038/s41598-025-30859-9.

## Introduction

Migraine is a common cause of disability worldwide, affecting daily functioning even in children^1,2^. The prevalence of primary headache in children has been reported to be 62%, with migraine accounting for 11% and tension-type headache for 17%^3^. The prevalence of headache among Japanese elementary and junior high school students has been reported to range from 49.4% to 59.8%^4,5^. These findings suggest that headache is a condition frequently encountered in educational settings.

Pediatric migraine affects not only school and social life but also parental work productivity^6^. Beyond headache pain, it is linked to psychosocial issues such as loneliness and depression, with unpredictable attacks disrupting daily routines and academic performance^7,8^. Early diagnosis and timely intervention are vital to improve outcomes and prevent chronification^8^.

Over-the-counter (OTC) medications are among the more accessible treatments for children with migraine. A large-scale study reported that 17.6% of adolescents used OTC analgesics at least once a week, primarily for headache, with higher usage in those with anxiety and depression^9^. Many pediatric patients are assumed to self-manage with OTC drugs without appropriate medical care, which may increase the risk of medication-overuse headache (MOH)^10^.

In Japan, where nearly all children attend school, the frequency of headache consultations with school staff and subsequent medical visits is unclear. This study examined headache prevalence, coping strategies, school consultations, and medical visits in one municipality.

## Results

### Prevalence of headache and comparison by grade

**Fig. 1:**
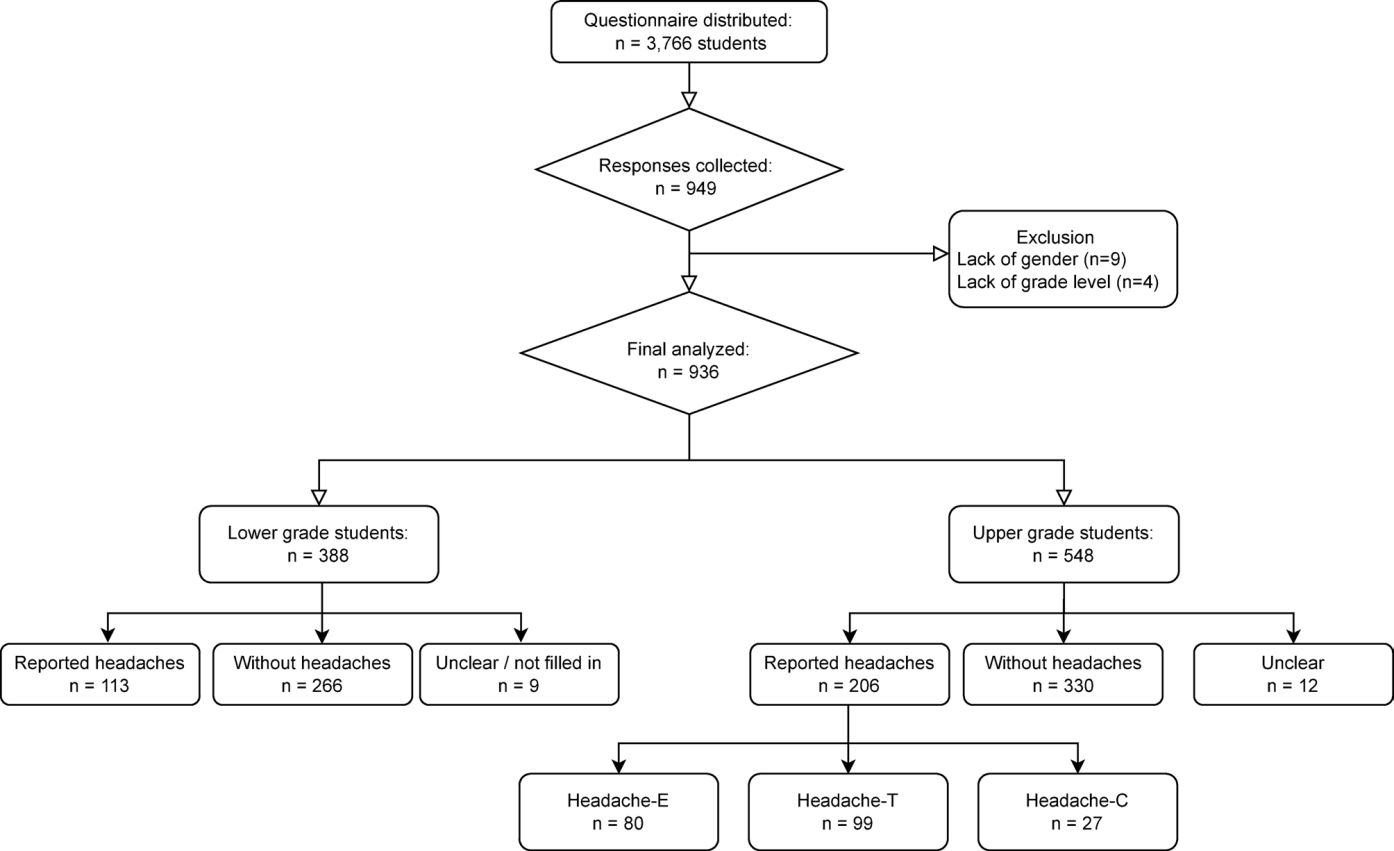
Flow chart. Flow chart showing the distribution of the 3766 distributed questionnaires, number of missing information on gender or school grade, and classification of participants into lower-grade and upper-grade groups. Abbreviations: Headache-E = Headaches that meet most of the diagnostic criteria for episodic migraine, Headache-T = Headaches that meet most of the diagnostic criteria for episodic tension-type headache, Headache-C = Headaches that meet most of the diagnostic criteria for chronic migraine or tension-type headache.

The survey was distributed to 3,766 students, and a total of 949 students responded to the survey. Responses with missing information on either gender or school grade (*n* = 13) were excluded from the analysis (Fig. 1).

A total of 936 participants were included in the final analysis (437 male, 491 female, 8 preferred not to answer), resulting in a response rate of 24.9%. Of these, 388 students were in the lower-grade group (200 male, 188 female, 0 preferred not to answer: 30.0%) and 548 were in the upper-grade group (237 male, 303 female, 8 preferred not to answer 22.2%).

In response to the question “Do you usually experience headache?”, 319 students (34.1%) answered “Yes” (113 lower-grade students [29.1%, CI: 25.0%-34.0%] and 206 upper-grade students [37.6%, CI: 33.5%-41.8%]).

The prevalence by grade was as follows: 29.7% (41/138, CI: 22.2%-38.1%) in first grade, 27.7% (38/137, CI: 20.4%-36.0%) in second grade, 30.1% (34/113, CI: 21.8%-39.4%) in third grade, 32.1% (36/112, CI: 23.6%-41.6%) in fourth grade, 32.3% (40/124, CI: 24.1%-41.2%) in fifth grade, 32.7% (32/98, CI: 23.5%-42.9%) in sixth grade, 38.0% (27/71, CI: 26.8%-50.3%) in seventh grade (junior high year 1), 50.5% (49/97, CI: 40.2%-60.8%) in eighth grade, and 47.8% (22/46, CI: 32.9%-63.1%) in ninth grade (Table 1).

**Table 1:** Prevalence of headache by grade in Japanese elementary and junior high school.

Grade	Total	Headache (%)	Headache-E (%)	Headache-T (%)	Headache-C (%)
1st	138	41 (29.7)	n.d.	n.d.	n.d.
2nd	137	38 (27.7)	n.d.	n.d.	n.d.
3rd	113	34 (30.1)	n.d.	n.d.	n.d.
4th	112	36 (32.1)	10 (8.9)	25 (22.3)	1 (0.9)
5th	124	40 (32.3)	15 (12.1)	19 (15.3)	6 (4.8)
6th	98	32 (32.7)	10 (10.2)	20 (20.4)	2 (2.0)
7th	71	27 (38.0)	12 (16.9)	11 (15.5)	4 (5.6)
8th	97	49 (50.5)	23 (23.7)	14 (14.4)	12 (12.4)
9th	46	22 (47.8)	10 (21.7)	10 (21.7)	2 (4.3)

n.d.: not determined.

The total number of students with headache is shown. For upper-grade students, the number and percentage of those classified as having Headache-E, Headache-T and Headache-C are also indicated. Abbreviations: Headache-E = Headaches that meet most of the diagnostic criteria for episodic migraine, Headache-T = Headaches that meet most of the diagnostic criteria for episodic tension-type headache, Headache-C = Headaches that meet most of the diagnostic criteria for chronic migraine or tension-type headache.

The count of headaches that meet most of the diagnostic criteria for episodic migraine (Headache-E) cases is as follows: 10 in fourth grade (8.9%), 19 in fifth grade (15.3%), 13 in sixth grade (13.3%), 14 in seventh grade (19.7%), 30 in eighth grade (30.9%), and 11 in ninth grade (23.9%) (Table 1).

### Headache managements and associated daily life

To assess headache-related management and its effect on school attendance, we analyzed medication use and school absence. In response to the question, “Have you ever taken medication for a headache?”, 43.4% of lower-grade students and 70.4% of upper-grade students answered “yes” (Table 2). Regarding absenteeism due to headaches, 27.4% of lower-grade students and 53.9% of upper-grade students reported having missed school because of headaches (Table 2).

**Table 2:** Impact of headache on daily life and its management by children.

	Low grade students, *n* (%)			Upper grade students, *n* (%)		
	Yes	No	Others*	Yes	No	Others*
Use of medication	49 (43.4)	61 (54.0)	3 (2.7)	145 (70.4)	59 (28.6)	2 (1.0)
School absence	31 (27.4)	81 (71.7)	1 (0.9)	111 (53.9)	93 (45.1)	2 (1.0)
Consultation for teachers	44 (38.9)	67 (59.3)	2 (1.8)	102 (49.5)	100 (48.5)	4 (1.9)
Consultation for doctors	26 (23.0)	86 (76.1)	1 (0.9)	69 (33.5)	129 (62.6)	8 (3.9)

*“Others” refer to cases where no answer was given or the answer was unclear.

As for consultations about headaches, 38.9% of lower-grade students had spoken with school staff, and 23.0% had visited a medical facility. In the upper-grade group, these figures were higher, with 49.5% having consulted school personnel and 33.5% having sought medical care (Table 2).

Next, headache frequency was examined among the 319 students who responded that they “usually experience headaches.” The most commonly reported frequency was once per month (*n* = 109, 34.2%), followed by 4–5 times per month (*n* = 67, 21.0%) and 2–3 times per year (*n* = 60, 18.8%) (Supplementary Fig. 1).

In the lower-grade students, the majority reported experiencing headaches 2–3 times per year or once per month. In contrast, students in the upper-grade students tended to report higher frequencies, ranging from once per month to 4–5 times per week.

Among the 206 upper-grade students who reported experiencing headaches regularly, the age of onset was collected via open-ended responses. The most frequently reported age of onset was 10 years old (typically corresponding to fourth grade). Reported onset ages ranged broadly from 4 to 14 years (Supplementary Fig. 2).

### The comparison among headache subgroups

Among the 206 upper-grade students who reported having headaches, participants were classified into three groups (Headache-E, Headaches that meet most of the diagnostic criteria for episodic tension-type headache (Headache-T), Headaches that meet most of the diagnostic criteria for chronic migraine or tension-type headache (Headache-C)) for comparative analysis.

Significant differences were observed among the three groups in term of consultation for doctor (*p* < 0.001) and school absence(*p* = 0.004) (Table 3). Headache frequency differed among the three groups (*p* < 0.001).

**Table 3:** Comparison of clinical features and headache-related experiences between the headaches that Meet most of the diagnostic criteria for episodic migraine (Headache-E), headaches that Meet most of the diagnostic criteria for episodic tension-type headache (Headache-T) and headaches that Meet most of the diagnostic criteria for chronic migraine or tension-type headache (Headache-C). Headache frequency refers to the number of headache days reported over the past three months. A p-value of < 0.05 was considered statistically significant. Different superscript letters indicate statistically significant differences between groups (Bonferroni-adjusted *p* < 0.05). Group sharing the same letter are not significantly different.

	Headache-E	Headache-T	Headache-C	*p* value
n	80	99	27	
M/F/O	30/49/1	35/63/1	7/19/1	0.524
Number of headache days in past three months (Mean ± S.E.)	5.92 ± 0.74^b^	4.1 ± 0.45^b^	37.88 ± 5.90^a^	< 0.001
Medication (Yes, %)	61 (76.3)	62 (62.6)	22 (81.5)	0.087
Consultation for teachers (Yes, %)	42 (52.5)	43 (43.4)	17 (63.0)	0.241
Consultation for doctors (Yes, %)	33 (41.3)^a^	17 (17.2)^b^	19 (70.4)^b^	< 0.001
Absent (Yes, %)	53 (66.3)^b^	41 (41.4)^b^	17 (63.0)^a^	0.004

Different superscript letters indicate significant differences between groups (Dunn’s test with Bonferroni correction, *p* < 0.05).

Post hoc Dunn’s test with Bonferroni correction showed that the Headache-C group had significantly more headache days than both the Headache-E and Headache-T groups (both *p* < 0.001), with no significant difference between Headache-E and Headache-T. Bonferroni-corrected pairwise comparisons revealed that the Headache-C group had a significantly higher rate of school absence compared to both the Headache-E and Headache-T groups. In contrast, students with Headache-E were more likely to consultation for doctor than those with Headache-T, while no significant difference was observed between the Headache-C group and the other two groups in terms of consultation behavior.

Headache triggers were also investigated among upper-grade students who reported regular headaches, using a multiple-choice format (multiple responses allowed). The most frequently reported trigger was stress (*n* = 98, 47.6%), followed by weather changes (*n* = 96, 46.6%) and lack of sleep (*n* = 85, 41.3%) (Table 4). In the Headache-T group, no trigger was reported by over 40% of students, whereas in the Headache-E and Headache-C groups, some (e.g., stress, weather) exceeded 50%.

**Table 4:** Reported headache triggers among upper-grade students who regularly experience headaches.

*n* (%)	All *n* = 206	Headache-E *n* = 80	Headache-T *n* = 99	Headache-C *n* = 27	*p* value
Stress	98 (47.6)	39 (48.8)	37 (37.4)	22 (81.5)	< 0.001
Weather	96 (46.6)	46 (57.5)	34 (34.3)	16 (59.3)	0.003
Lack of sleep	85 (41.3)	33 (41.3)	38 (38.4)	13 (51.9)	0.452
Study	47 (22.8)	18 (22.5)	18 (18.2)	11 (40.7)	0.046
Menstruation	46 (22.3)	20 (25.0)	15 (15.2)	11 (40.7)	0.014
Sound	30 (14.6)	11 (13.8)	11 (11.1)	8 (29.6)	0.052
Shoulder stiffness	30 (14.6)	16 (20.0)	8 (8.1)	6 (22.2)	0.038
Exercise	27 (13.1)	12 (15.0)	12 (12.1)	3 (11.1)	0.806
Light	27 (13.1)	15 (18.8)	6 (6.1)	6 (22.2)	0.014
Smell	26 (12.6)	14 (17.5)	6 (6.1)	6 (22.2)	0.020
Oversleeping	25 (12.1)	8 (10.0)	13 (13.1)	4 (14.8)	NA
Unclear	10 (4.8)	2 (2.4)	7 (7.1)	1 (3.7)	NA
Use of smartphones	8 (3.8)	3 (3.7)	2 (2.0)	3 (11.1)	NA
Fatigue	5 (2.4)	0 (0)	5 (5.1)	0 (0)	NA
Foods	4 (1.9)	3 (3.7)	0 (0)	1 (3.7)	NA
Lack of water	3 (1.4)	0 (0)	3 (3.0)	0 (0)	NA

NA: not applicable. P-value not calculated due to small sample size.

## Discussion

In our study revealed for key findings: (1) the prevalence of headache was 29.2% in the lower-grade students and 37.3% in the upper-grade students, (2) the most frequently reported age of onset for headaches was 10 years old, (3) Students in the Headache-C group experienced significantly more headache days and greater disruption to school life compared to other groups, (4) common headache triggers such as stress, weather changes, and lack of sleep – were similar to those reported in adults.

A meta-analysis reported migraine prevalence in children and adolescents at 11%, rising from 5% in younger children to 15% in adolescence^3^. Onset typically occurs around ages 10–13, consistent with domestic data^11^. Katsuki et al.^12^ noted peaks at 6–8 and 15–17 years. Tension-type headaches are more common in elementary school, while migraines predominate in junior high^13^. Both increase after puberty, likely linked to hormonal changes^14^. In this study, most junior high students with headaches had migraines, supporting this association. While lower grades showed minimal sex differences, upper grades had more females. Hormonal changes, particularly those related to menstruation, are known triggers for migraine in adolescent girls. Therefore, providing education on menstruation and its possible links to headache may help promote awareness, early self-management, and timely access to appropriate treatment.

This study found a greater headache burden in students with Headache-C, who reported significantly more headache days than those with Headache-T. Additionally, 27 students met criteria for Headache-C, averaging 37.9 headache days over three months. Consistent with prior studies, headaches were linked to school absenteeism^15^, yet medical consultation rates remained low (23.7% lower grades; 33.2% upper grades), despite 39–50% reporting medication use. Notably, 15.4% of students aged ≥ 10 used pain medication several times a week or daily, raising concerns for MOH. Many relied on OTC drugs without diagnosis, consulting school staff more than physicians. These findings highlight the need for stronger collaboration between schools and healthcare providers to improve headache management.

Stress and sleep disturbances are the most common migraine triggers in Asian populations^16^, aligning with our study, where both were frequently cited across age groups. About 40% reported sleep deprivation regardless of group, reinforcing its role as a general pediatric headache trigger. Sleep problems are closely linked to migraine severity^17^, highlighting the importance of good sleep hygiene and screen time management. A Kuwaiti study found 99.7% of children owned smart devices, with 59.9% overusing them, often at night^18^; overuse is linked to headaches and poor sleep^19,20^. Non-pharmacological approaches, including limiting screen use before bedtime, are essential. In Japan, triggers such as hunger, sunlight, shoulder stiffness, weather, anxiety, and schoolwork were significantly associated with migraine^5^. In our study, stress was reported by 81.5% of the Headache-C group, and weather was common in both Headache-E and Headache-C groups, suggesting shared triggers.

This study has several limitations. The most important limitation of this study is that it did not involve clinical examinations by headache specialists. And we did not use the standard questionnaire like MIDAS. Although this was noted in the Methods section, the classification of headache types was based solely on self-reported symptoms and should not be interpreted as definitive clinical diagnoses. The terms used in this study, such as “Headache that meet most of the diagnostic criteria for episodic migraine (Headache-E)”, “Headaches that meet most of the diagnostic criteria for episodic tension-type headache (Headache-T)”, and “Headaches that meet most of the diagnostic criteria for chronic migraine or tension-type headache (Headache-C)”, are symptom-based categorizations intended for analytical purposes only. Next to, the low response rate (24.9%) may limit generalizability and introduce self-selection bias, though the rate was higher among lower-grade students (30.3%). In addition, there is a possibility of participation bias, as children or parents with greater interest in headaches or health issues may have been more likely to respond, while those less concerned may have been underrepresented. Online-only responses may have affected participation due to parental involvement and internet access. Future studies should consider more accessible survey methods. The data were collected via self-reported online questionnaires, which did not require login or personal identifiers. Therefore, it is possible that multiple submissions by the same individual could have occurred, although we instructed respondents to submit only once. This may have introduced some degree of overreporting or duplication bias, which should be taken into account when interpreting the results. As all questionnaire items were optional and some participants did not respond to certain questions, there is a possibility of response bias arising from selective answering.

Second, the questionnaire lacked items on headache duration and was not formally validated. Students were classified as having migraine without evaluating duration (ICHD-3 Criterion B) or the required number of attacks (Criterion A), possibly leading to misclassification. Despite this, students identified as migraine cases showed higher headache frequency and school disruption, aligning with prior research. Future surveys should include headache duration and frequency for more accurate classification. Lastly, this study could not diagnose chronic migraine or tension-type headache due to the questionnaire’s limitations. Another limitation is the relatively small sample size, which may limit the generalizability of the findings. Finally, this study did not include multivariate analyses to adjust for potential confounding factors such as gender or school grade, which may influence the relationship between headache subtypes and reported triggers. Future studies with larger sample sizes should consider stratified or multivariable approaches to clarify these associations.

In conclusion, this study examined headache prevalence and characteristics among students in a single municipality, focusing on consultation behaviors and school impact. Students with migraine-like features had more frequent headaches and higher absenteeism. Fewer students consulted physicians than school staff, highlighting a gap between education and healthcare. Raising awareness among teachers, parents, and students, and fostering collaboration, is crucial for early detection and intervention.

## Methods

### Survey protocol

Participants were students enrolled in seven public elementary schools and three public junior high schools in Ami-town, Ibaraki Prefecture, Japan (population approximately 50,000 as of February 2025). We targeted all 10 public elementary and junior high schools in the town, representing the entire population of school-aged children attending public schools in the area. The survey was conducted over a 31-day period, from February 12 to March 14, 2025. Responses were collected online using Google Forms. The survey was distributed via a mobile application installed on guardians’ smartphones, as well as by providing a QR code on printed materials. For younger children who may have had difficulty completing the questionnaire on their own, it was noted that guardians were encouraged to assist with responses. All questionnaires were anonymous and consisted solely of open-ended and non-identifiable items. Participation was considered to imply consent, as stated in the research cooperation statement.

This study was approved by the Ethics Committee of Ibaraki Prefectural University of Health Sciences (approval date: January 9, 2025; approval numbers: e484, e490) and was conducted in accordance with the Declaration of Helsinki. In addition, permission for cooperation was obtained from the Ami Town Board of Education and the principals of all participating schools. The survey was distributed via a school communication app installed on guardians’ smartphones, as well as by providing a QR code on printed materials. Informed consent was considered obtained from a parent or legal guardian through completion of the questionnaire. Prior to distribution, an information letter explaining the study was sent to all guardians through the school communication app. This procedure was reviewed and approved by the ethics committee.

### Questionnaire items

The questionnaire was developed in two versions: one for grades 1–3 of elementary school (hereafter referred to as the “lower-grade student questionnaire”) and one for grades 4–6 of elementary school and grades 1–3 of junior high school (hereafter referred to as the “upper-grade student questionnaire”) (Supplementary Table 1).

The lower-grade student questionnaire included 10 items. Q1 and Q2 referred to gender (male, female, or prefer not to answer) and current grade, respectively. Q3 asked whether they usually experience headaches (yes, no, or unknown), and only those who answered “yes” proceeded to Q4–Q9. Q4 assessed headache frequency using options ranging from “once a year” to “every day.” Q5 asked about headache type (dull/tight, throbbing, or unknown). Questions 6 to 9 addressed medication use, school absence, medical consultation, and daily life impact, with yes/no/unknown responses. All questions were optional.

The upper-grade questionnaire comprised 20 items. Q1–3 and Q5 matched the lower-grade version. Q4 (onset) and Q6 (headache frequency in the past three months) were short-answer, while Q7 assessed pain location (unilateral, bilateral, or variable). Q8–12 and Q16–18 used yes/no/unknown responses. Q13 covered headache type (dull, throbbing, or feel like lying down), and Q15 asked for medication frequency (free-text). A multiple-choice item listed triggers such as sleep issues, stress, light, sound, weather, study, menstruation, food, and shoulder stiffness. No items were mandatory. The questionnaire was based on the International Classification of Headache Disorders, 3rd edition (ICHD-3) criteria^14^, and students meeting criteria C and D for migraine without aura were categorized as Headache-E. Those who met most of the diagnostic criteria for episodic tension-type headache were classified as having Headache-T. In addition, students who reported experiencing headaches “daily” or “several times a week” in Q5 (headache frequency) were categorized as having Headache-C. Since this was an anonymous questionnaire survey and participants did not undergo clinical evaluation by headache specialists, headache subtypes were categorized based on self-reported symptoms and should not be interpreted as clinical diagnoses.

### Statistical analysis

Ordinal variables were presented as frequencies and percentages. Confidence intervals (CI) were calculated using R software version 4.3.2. For comparisons between the groups, Chi-square test or the Kruskal–Wallis test was used, as appropriate. In the ordinal scale, Dunn’s tests with Bonferroni correction was used as a post hoc test. All statistical analyses were performed using SPSS version 30.00 and GraphPad Prism 10, with a significance level set at *p* < 0.05.

A post hoc power analysis was conducted using G*Power 3.1 software. The analysis revealed sufficient statistical power for both the chi-square test (1 – β = 0.978, w = 0.3) and the Kruskal–Wallis test (1 – β = 0.976, f = 0.3), based on a total sample size of 206 and three groups.

## Supplementary Information

Below is the link to the electronic supplementary material.

Supplementary Material 1

## Data Availability

The datasets generated and/or analyzed during the current study are available from the corresponding author on reasonable request.

## Abbreviations

CI: Confidence interval
EM: Episodic migraine
F: Female
Headache-E: Headaches that meet most of the diagnostic criteria for episodic migraine
Headache-T: Headaches that meet most of the diagnostic criteria for episodic tension-type headache
Headache-C: Headaches that meet most of the diagnostic criteria for chronic migraine or tension-type headache
ICHD-3: International Classification of Headache Disorders, 3rd edition
M: Male
MOH: Medication-overuse headache
NA: Not applicable
OR: Odds Ratio
OTC: Over-the-counter
