# Supplementary material for: Headache prevalence and impact among school-aged children in a Japanese town: the AMI-GRAINES study
Source: Sci Rep. 2025 Dec 3;16:1144. doi: 10.1038/s41598-025-30859-9 (PMC12789055; doi:10.1038/s41598-025-30859-9)
Supplement: Supplementary file 1 — Supplementary Material 1 [file 41598_2025_30859_MOESM1_ESM.docx]

Supplementary table.1 The questionnaire items

| Lower-grade students Questionnaire | |  |
| --- | --- | --- |
| Q1 | Gender | |
| Q2 | Grade level | |
| Q3 | Do you usually experience headaches? | |
| Q4 | Headache frequency Please select one. | |
| Q5 | What kind of pain? Please select one. | |
| Q6 | Have you ever taken medication for a headache? | |
| Q7 | Have you ever talked to a school teacher about your headache? | |
| Q8 | Have you ever been absent from school because of a headache? | |
| Q9 | Have you ever visited a doctor because of a headache? | |
| Q10 | If there is anything that troubles you because of your headaches, please write about it below. | |
| Upper-grade students Questionnaire | |  |
| Q1 | Gender | |
| Q2 | Grade level | |
| Q3 | Do you usually experience headaches? | |
| Q4 | When was the onset of your headaches? | |
| Q5 | Headache frequency Please select one. | |
| Q6 | How many days have you had headaches in the past three months? | |
| Q7 | What are the characteristics of your headaches? Please select one. | |
| Q8 | Is your headache moderate to severe in intensity? | |
| Q9 | Does your headache get worse with routine physical activities (such as walking or climbing stairs)? | |
| Q10 | Do your headaches come with nausea or vomiting? | |
| Q11 | Does light bother you when you have a headache? | |
| Q12 | Are you sensitive to sound when you have a headache? | |
| Q13 | Which of the following best describes your headache? | |
| Q14 | Have you ever taken medication for a headache? | |
| Q15 | **How often do you take pain medicine when you have a headache?**(e.g., about ___ times per month) | |
| Q16 | Have you ever been absent from school or club activities because of a headache? | |
| Q17 | Have you ever talked to a school teacher about your headache? | |
| Q18 | Have you ever visited a doctor because of a headache? | |
| Q19 | Please select the factors that may trigger your headaches. (Multiple answers allowed) | |
| Q20 | If there is anything that troubles you because of your headaches, please write about it below. | |


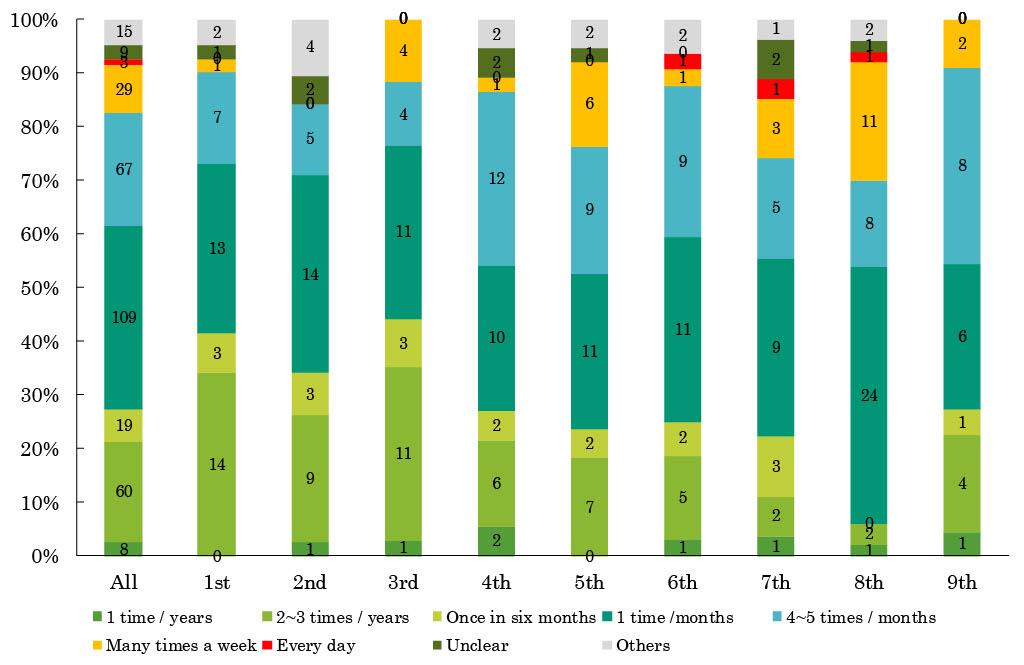


Supplementary figure 1. Frequency of headache by grade. *Those who did not fill (n=2) are excluded from the figure.*

Supplementary figure 2. **Self-reported age of headache onset among upper-grade students with regular headaches.** The most frequently reported age of onset was 10 years, with responses ranging from 4 to 14 years.
